# Sampling intraspecific variability in leaf functional traits: Practical suggestions to maximize collected information

**DOI:** 10.1002/ece3.3617

**Published:** 2017-11-21

**Authors:** Francesco Petruzzellis, Chiara Palandrani, Tadeja Savi, Roberto Alberti, Andrea Nardini, Giovanni Bacaro

**Affiliations:** ^1^ Department of Life Sciences University of Trieste Trieste Italy; ^2^ Department of Agricultural, Food, Environmental and Animal Sciences University of Udine Udine Italy

**Keywords:** osmotic potential, PERMANOVA, precision, *Quercus ilex*, specific leaf area, variance partitioning

## Abstract

The choice of the best sampling strategy to capture mean values of functional traits for a species/population, while maintaining information about traits’ variability and minimizing the sampling size and effort, is an open issue in functional trait ecology. Intraspecific variability (ITV) of functional traits strongly influences sampling size and effort. However, while adequate information is available about intraspecific variability between individuals (ITV_BI_) and among populations (ITV_POP_), relatively few studies have analyzed intraspecific variability within individuals (ITV_WI_). Here, we provide an analysis of ITV_WI_ of two foliar traits, namely specific leaf area (SLA) and osmotic potential (π), in a population of *Quercus ilex* L. We assessed the baseline ITV_WI_ level of variation between the two traits and provided the minimum and optimal sampling size in order to take into account ITV_WI_, comparing sampling optimization outputs with those previously proposed in the literature. Different factors accounted for different amount of variance of the two traits. SLA variance was mostly spread within individuals (43.4% of the total variance), while π variance was mainly spread between individuals (43.2%). Strategies that did not account for all the canopy strata produced mean values not representative of the sampled population. The minimum size to adequately capture the studied functional traits corresponded to 5 leaves taken randomly from 5 individuals, while the most accurate and feasible sampling size was 4 leaves taken randomly from 10 individuals. We demonstrate that the spatial structure of the canopy could significantly affect traits variability. Moreover, different strategies for different traits could be implemented during sampling surveys. We partially confirm sampling sizes previously proposed in the recent literature and encourage future analysis involving different traits.

## 1 INTRODUCTION

Plant traits are defined as any morphological, physiological, or phenological features measurable at the individual level, from the cell to the whole‐organism (Violle et al. 2007). In the last decades, plant functional traits have been widely included in trait‐based studies, because they impact fitness indirectly via effects on growth, reproduction, and survival (Violle et al. 2007), and reflect the trade‐offs among different physiological and ecological functions (Díaz & Cabido, 2001; Lavorel et al., 2007; McIntyre, Díaz, Lavorel, & Cramer, 1999). A wide range of ecological issues can be conveniently addressed using a functional trait approach. For example, functional diversity helps addressing questions about determination of ecosystems level processes (Díaz & Cabido, 2001; Escudero & Valladares, 2016) or to disentangle processes underlying invasions by alien species as well as invasion resistance (Drenovsky et al., 2012; Funk, Cleland, Suding, & Zavaleta, 2008). Furthermore, assessment of species traits can be used in modeling vegetation changes under different environmental pressure (Hobbs, 1997; Noble & Gitay, 1996) and in managing ecosystem services (Lavorel & Garnier, 2002).

The number of studies based on the analysis of functional traits has steadily increased in recent years. Yet, there are still some critical issues to be solved, related to the cost‐benefits of different sampling strategies to capture the variability of functional traits between and within communities. A very actual debate in “trait‐based ecology” is focused on the importance and relative magnitude of interspecific (BTV, B stands for between species) and intraspecific variability (ITV) (Albert et al., 2010). While sources and effects of BTV on functional trait‐based studies have been widely investigated (Diaz et al., 2004; Wright et al., 2005), the contribution of ITV to the total variability of a trait has been underestimated (Violle et al., 2012). ITV is defined as the overall variability of trait values and trait syndromes (set of trait values including trait trade‐offs) expressed by individuals within a species (Albert, Grassein, Schurr, Vieilledent, & Violle, 2011). The commonly accepted paradigm is that the BTV is much larger than ITV (Albert et al., 2011), leading to the so‐called ITV<BTV assumption (Garnier et al., 2001; Wilson, Thompson, & Hodgson, 1999). Therefore, ITV has been often considered negligible. Recently, a growing number of studies has shown that this assumption is not always correct (Albert, 2015; Albert et al., 2010; Siefert et al., 2015) and provided frameworks and suggestions on procedures to account for ITV. For example, Siefert et al. (2015) demonstrated that different traits have different ITV magnitude and that ITV must be taken into account when specific leaf area (SLA), leaf dry matter content (LDMC), or leaf chemical traits are included in functional traits‐based studies. Albert et al. (2011) also proposed a framework to assess when and how ITV should be taken into account.

According to Albert et al. (2011), ITV comprises three levels: (1) between population level variability (ITV_POP_); (2) between individual variability (ITV_BI_); (3) within individual variability (ITV_WI_). Large attention had been dedicated to the first two levels, while the latter had been scarcely investigated in a rigorous way. ITV_WI_ is defined as the feature of traits that vary within individuals (Albert et al., 2011) and could arise due to genotypic, phenotypic, or ontogenetic processes (Messier, McGill, & Lechowicz, 2010; Valladares et al., 2014). In particular, significant micro‐environmental gradients can occur even within the canopy of single trees (Niinemets, 2016), thus affecting leaf traits values magnitude and distribution within a single individual.

Leaves display a series of attributes that are linked to specific functions (functional leaf traits) and/or show responses to biotic and abiotic stress factors (stress response traits), which can be subdivided into: (1) morphological traits; (2) chemical traits; (3) physiological traits; (4) syndromes. The analysis of functional leaf traits is a useful tool for tree species and provenance phenotyping, due to the adaptation of trees to environmental stress (Gratani, Meneghini, Pesoli, & Crescente, 2003). Additionally, functional leaf traits can be used as response factor in long term and large spatial scales surveys of forest and crops conditions (Apgaua et al., 2017; Martin et al., 2017). Indeed, leaf sampling and analysis is a tool applied in research projects and monitoring programs (Rautio, Fürst, Stefan, Raitio, & Bartels, 2010), but sampling an adequate numbers of leaves can be a difficult, time‐consuming, and costly task, because of horizontal structure of forest making samples difficult to access.

Under this perspective, the choice of traits and the number of replicates to capture the mean value (and the associated variability) of leaf traits for target populations remains an issue only partially solved in trait‐based ecology. In this perspective, standardized protocols are mandatory to compare the variability of different studies and to perform general inferences on ecological mechanisms. In the last 10 years, multiple handbooks of protocols (e.g., Cornelissen et al., 2003; Pérez‐Harguindeguy et al., 2013) have listed the most used plant traits and proposed sampling standards for each of them (i.e., how to measure traits, the minimum and preferred number of replicates, etc.). Moreover, these protocols support decisions for the selection of an appropriate sample size depending on the purpose of the study and on the desired precision level. Before protocols application, however, optimal sampling parameters should be assessed on the basis of the extent of ITV in the study area (Cornelissen et al., 2003). Here, we provide an analysis of ITV_WI_ of two foliar traits, one “functional” (SLA) and one “mechanistic” (osmotic potential or π), in a population of *Quercus ilex* L. (Holm oak). According to Brodribb (2017), mechanistic traits are characterized by a clear association with a specific physiological function, while general functional traits (such as SLA) rather represent a “syndrome” that can be associated to different physiological functions and associated trade‐offs. Despite the deeper physiological insights provided by mechanistic traits than by functional ones, they are more difficult to measure (in terms of costs and time) and, therefore, are scarcely included in trait‐based studies. Consequently, very little is known about mechanistic traits variability, while several studies investigated variation of soft traits at different ecological scales (Messier et al., 2010). Anyway, recent studies have proposed new techniques for time‐ and cost‐effective estimation of different mechanistic traits (e.g., Bartlett, Scoffoni, Ardy et al. (2012) for osmotic potential, or Skelton, Brodribb, and Choat (2017) for vulnerability to xylem embolism).

Based on an intensive spatially explicit sampling of the two described foliar traits, this study is aimed at: (1) assessing the baseline ITV_WI_ level of variation between the two traits; (2) assessing the minimum and optimal sampling size in order to take into account ITV_WI_; (3) comparing sampling optimization outputs with those previously proposed in the literature; (4) proposing practical advises and sampling scenarios useful for ecologists and biologists to plan traits sampling campaigns.

## 2 MATERIALS AND METHODS

### 2.1 Study area and sampling design

This study was performed in the Cernizza woodland (45°46′37.4″ N, 13°45′21.2″ E), an area located in the Karst region (NE Italy) at 40 m a.s.l. The climate is humid temperate with higher precipitation in October‐November and a relatively dry spell in August‐September (Furlanetto, 2003). The woodland hosts typical Mediterranean evergreen species, including *Quercus ilex*,* Phyllirea latifolia*,* Osyrys alba*,* Smilax aspera*,* Ruscus aculeatus,* and *Rubia peregrina* (Del Favero & Poldini, 1998).

**Figure 1:**
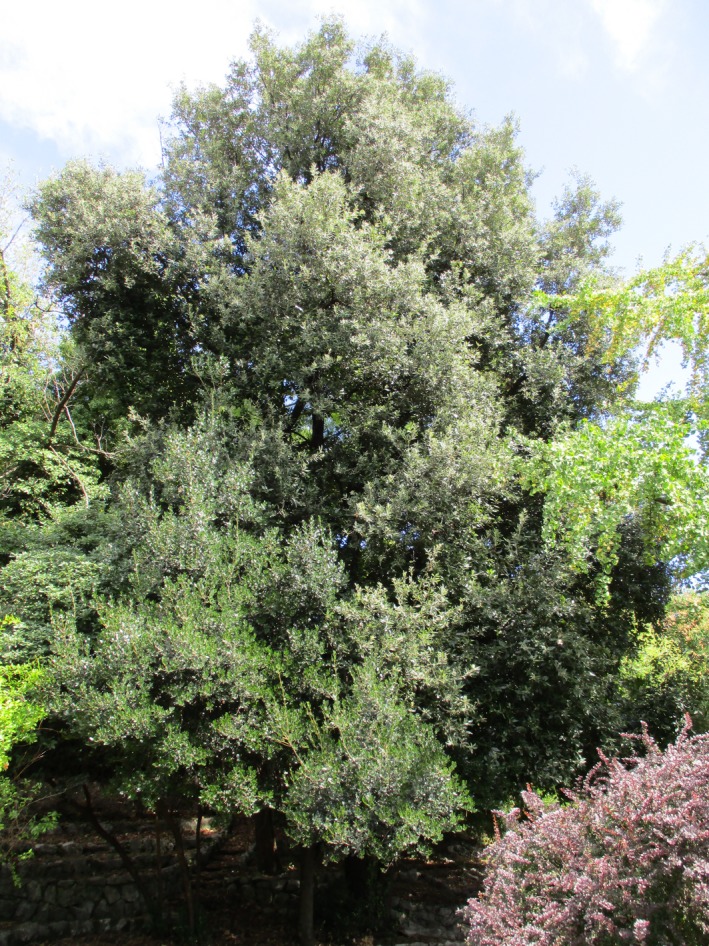
Individual of *Quercus ilex* growing in the study area

*Quercus ilex* (Figure 1) is the dominant woody species and occurs in different environmental conditions due to the heterogeneous substrate of the study area (Nardini et al., 2016). For these reasons, we choose *Q. ilex* as the study species. We first identified three areas (0.6 ha each), approximately 100 m apart from each other and characterized by the highest density of *Q. ilex* according to a recent map by Furlanetto (2003) (Fig. S1). We extrapolated the centroid of each area using the software Quantum Gis (v. 2.12.0 – Lyon. QGIS Development Team, 2015) and the *Q. ilex* individual closest to the centroid represented the center of a 10 × 10 m quadrat. We sampled every individual of *Q. ilex* with trunk diameter at breast height (DBH) ≥5 cm within each quadrat. In total, we sampled 34 individuals from three quadrats.

The sampling procedure was designed in order to collect the highest possible (given time and cost constraints) number of leaves within all the individuals in each quadrat. Clearly, a complete census even of a single crown is unrealistic. Hence, we realized an intensive sampling effort consisting in 12 pairs of leaves for each individual. Specifically, we sampled 12 twigs from each individual and one leaf pair was selected from each twig. We sampled leaf pairs because the measurement of the selected traits implies samples destruction. With the aim to capture as much variability as possible within each considered individual, each tree was divided in two height classes (“a” from base to 2.5 m, “b” from 2.5 m to the top) and in two vertical strata (external = E, internal = I). A total of 6 leaf pairs were selected for each height class; three pairs of them were external leaves (E) while the other three were internal leaves (I). The exposure (north, south, east, west) of the leaf pairs along the canopy was randomly assessed according to the following scheme. We generated a random series of number from 0 to 360. When this number was between 45 and 135 (45 < x < 135), sampled leaf pairs should be exposed to east; when 135 < x < 225 to south; 225 < x < 315 to west and 315 < x < 45 to north. Such stratification was designed to assess the contribution of different leaf position in the canopy to the total variance of the traits, as micro‐environmental gradients within the canopy could affect leaf traits values (Niinemets, 2016). Twigs bearing leaf pairs were detached, wrapped in cling film, put in humid sealed plastic bags and stored in coolbags until measurements in the laboratory. A total of 408 leaves from 34 different individuals were sampled from all the three quadrats in three different days, two in December 2015 and one in January 2016. The number of individuals per sampling unit differed, as expected, depending on the number of different trees occurring in each quadrat (five individuals in the first quadrat; 17 individuals in the second quadrat; 12 individuals in the third quadrat).

### 2.2 Measurements of leaf traits

As mentioned above, SLA and π were measured for each leaf pair.

Specific leaf area was calculated as the ratio between fresh leaf area and its dry weight, and it is expressed in mm^2^/mg.

$$\text{SLA}=\left(\text{Leaf Area}\right)/\left(\text{Leaf Dry Weight}\right)\;\left[{\text{mm}}^{2}/\text{mg}\right]$$

Specific leaf area is generally considered a “soft” structural trait, well‐related with relative growth rate and photosynthetic rate (Poorter & Remkes 1990). Plants adapted to arid and poor‐nutrients habitats usually show thicker and smaller leaves with higher lifespan and lower values of SLA (Pérez‐Harguindeguy et al., 2013). Fresh leaves were scanned using a scanner, and leaf area was measured using the software ImageJ (Schneider, Rasband, & Eliceiri, 2012). Leaves were then put in the oven for 48 h at 72°C, and then, leaf dry weight was measured using an analytical balance.

The osmotic potential (π) is considered a mechanistic trait, requiring time‐consuming procedures and specific instruments for measurements (Cornelissen et al., 2003). The standard method for the measurement of π is based on the elaboration of leaf water potential isotherms (or pressure‐volume curves) as described by Tyree and Hammel (1972). Recently, Bartlett, Scoffoni, Ardy et al. (2012) proposed an alternative procedure to measure π using vapor‐pressure osmometry of freeze‐thawed leaf disks. We used this method, with some modifications from the protocol proposed by Bartlett, Scoffoni, Ardy et al. (2012). Fresh leaves (without the petiole) were roughly crumbled and sealed in cling film. Then, they were immersed in liquid nitrogen (LN_2_) for 2 min. Leaves (still sealed in cling film) were then ground to the smallest possible size and stored in sealed plastic bottles at −20°C until measurements. Finally, π was measured using a dew point potentiameter (Model WP4, Decagon Devices Inc., Pullman, WA, USA) within two weeks after samples preparation.

### 2.3 Partitioning of spatial variability

The first goal of our analysis was to assess the spatial variation of ITV of the two measured traits. Hence, we performed a partitioning of spatial variability of SLA and π. Specifically, we assessed the variation in the two traits across four hierarchical organizational levels, namely quadrat (3 levels, fixed factor), individual (34 levels, fixed nested within quadrat), height class (2 levels, h_class, fixed), and position within the canopy (2 levels, E/I, fixed) through a multivariate analysis of variance by permutation (PERMANOVA, Anderson, 2001; McArdle & Anderson, 2001). The following interaction terms were tested: quadrat*h_class, quadrat*E/I, h_class*E/I, individual (quadrat)*h_class, individual (quadrat)*E/I, quadrat*h_class*E/I and individual(quadrat) *h_class*E/I. Euclidean distance was used as distance in this analysis. The percentage of variance explained by each factor was obtained by dividing the sum of squares (SS) calculated on the differences of distances from centroid of each factor by the total sum of squares.

The components of multivariate variance were tested for statistical significance with respect to 999 permutations of residuals under a reduced model (Anderson, 2001), using an a priori chosen significance level of α = .05.

The analysis was computed using software PRIMER (Clarke & Gorley, 2006) including the add‐on package PERMANOVA+6 (Anderson, Gorley, & Clarke, 2008).

### 2.4 Selection of most adequate sampling strategy and minimum and optimal sampling size

The second goal of our study was to provide the most adequate sampling strategy to measure SLA and π. We aimed at selecting the sampling strategy that minimizes the number of leaves and individuals (from here sampling size) needed to estimate traits values with desired precision and accuracy.

First, we calculated the minimum precision required to estimate the two traits adequately. Hence, we randomly resampled an increasing number of leaves (from 1 to 408) from the dataset, each time calculating the standard error (*SE*) as a precision measurement. The higher the *SE*, the lower the precision. We run the simulation 4,999 times for each number of leaves and, at the end, we were able to construct the relationship between *SE* and number of leaves considered. We assumed that the minimum desired precision corresponded to the *SE* value at the flex point (*SE*_min_) of this relationship. In fact, after this point, the relationship became linear and the slope slightly changed. The flex point was calculated using segmented function from package “SEGMENTED” (Muggeo, 2003) for R software (R Core Team, 2015).

**Figure 2:**
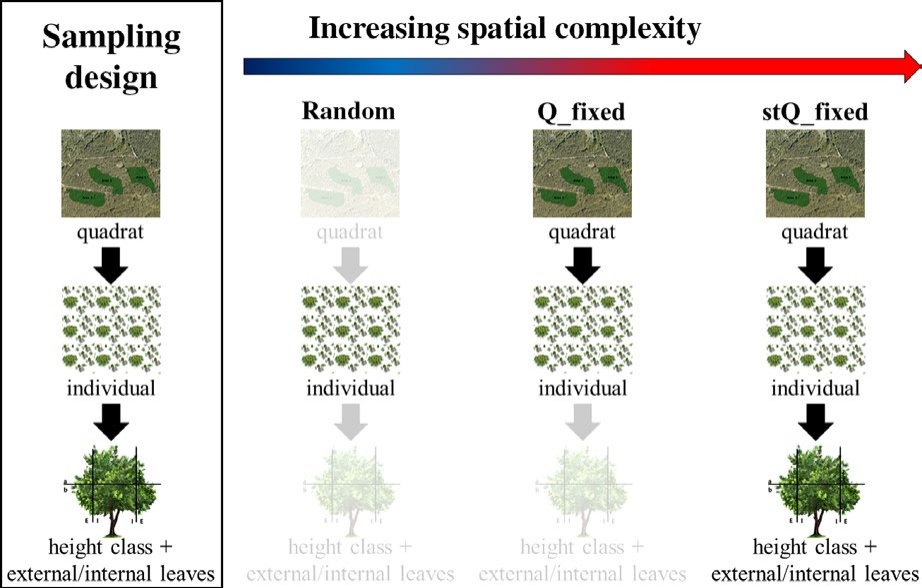
Illustration of sampling hierarchy (left boxes) and three examples of sampling strategies with different spatial complexity tested in this study

Secondly, we formulated 15 different sampling strategies adopting different selection criteria for the choice of leaves and individuals, constrained on the different levels of spatial organization (Figure 2, Table S1). RANDOM strategy was the less complex strategy, as leaves and individuals were sampled randomly, discarding all the spatial levels. In Q_fixed, an intermediate complex strategy, leaves were sampled randomly from an equal number of individuals within each quadrat. Finally, in stQ_FIXED, the most complex strategy, a fixed number of leaves were sampled from each canopy stratum (h_class and E/I) and from a fixed number of individuals per quadrat. All the strategies are resumed in Table S1. For each sampling strategy, we resampled an increasing number of leaves and individuals, starting from 1 leaves from 1 individual (“STRATEGY”_1_1) to 12 leaves from 34 individuals (“STRATEGY”_34_12). This analysis was conducted using mstage function from SAMPLING package (Tillé & Matei, 2016) for R software. In this way, we were able to test not only different sampling strategies, but also to select the minimum and optimal sampling size (e.g., number of individuals and leaves from each individual) to estimate SLA and π values. We simulated each possible combination of sampling size in each sampling strategy 4999 times, each time calculating SLA and π mean values, standard error (*SE*), and coefficient of variation (CV).

We then organized the selection of the most adequate sampling strategy and minimum and optimal sampling size in two steps. In the first step, we discarded all the strategies not meeting the following criteria:

1. Sampling strategy should produce a mean value of the two traits (SLA_strategy_, π_strategy_) that lies within the CI of the mean value calculated on the whole dataset (SLA_whole_data_, π_whole_data_);
2. Sampling strategy should have a mean *SE* equal or lower SE_min_, otherwise it was considered an unprecise strategy.

In the second step, we assessed the minimum and optimal sampling size of the sampling strategy selected as described above. More in detail, we measured the accuracy of each sampling size calculating the standardized deviations of the traits values estimated with each sampling size from SLA_whole_data_ and π_whole_data_:

$$S=\frac{|X_{m}-{\dot{X}}_{\mathrm{whole}\_data}|}{\mathrm{SD}({\dot{X}}_{\mathrm{whole}\_\mathrm{data}})},$$

where S = standardized deviation from mean value; *X*_m_ = π or SLA mean value of the corresponding sampling size; *Ẋ*_whole_data_ = π or SLA mean value of the original dataset; *SD*(*Ẋ*_whole_data_) = π or SLA standard deviation of the mean value. Sampling size that minimized the standardized deviation of the two traits was considered the most accurate.

At the end of this selection process, we were able to select the most adequate sampling strategy and minimum and optimal sampling size measuring their precision and accuracy.

## 3 RESULTS

**Figure 3:**
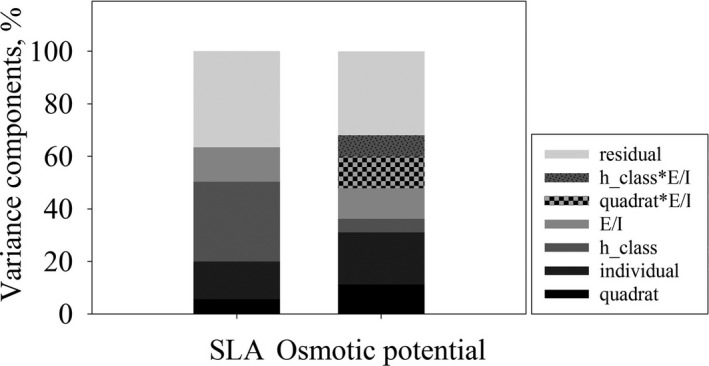
Estimated components of variance (expressed as percentages) in specific leaf area (SLA, mm^2^/mg) and osmotic potential (π, ‐MPa) values calculated for each factor

Factors expressing the spatial arrangement of leaves in the sampled areas, namely quadrat, individual, height class (h_class), and external/internal leaves (E/I), significantly affected variability of SLA and π (Table 1), and explained ~64% of the total variance in SLA measurements and ~60% of the total variance in π (Figure 3). Factors related to the spatial structure of the canopy (h_class, E/I and their interactions with other variables) accounted for 43.4% of the total variance of SLA, while they explained ~23% of the total variance of π. On the other hand, factors related to the distribution of individuals (quadrat, individual and their interactions with other variables) accounted for ~20% of the variance of SLA, while they explained 43.2% of the total variance of π.

**Table 1:** Results of PERMANOVA analysis of canopy structure variability at each spatial scale (quadrat, individuals within quadrats, height classes, external/internal leaves)

	Source	*df*	SS	MS	Pseudo‐*F*	*p* (perm)
SLA	Quadrat	2	4.67	2.33	3.72	**.033**
	h_class	1	69.77	69.77	111.14	**.001**
	E/I	1	9.94	9.94	15.84	**.001**
	individual (quadrat)	31	56.68	1.83	2.91	**.001**
	quadrat*h_class	2	0.21	0.11	0.17	.842
	quadrat*E/I	2	1.01	0.50	0.80	.454
	h_class*E/I	1	0.38	0.38	0.61	.440
	individual (quadrat)*h_class_	31	21.84	0.70	1.12	.263
	individual (quadrat)*E/I	31	22.84	0.74	1.17	.260
	quadrat*h_class*E/I	2	.49	1.74	2.78	.065
	individual(quadrat)*h_class*E/I	31	18.19	0.59	0.93	.590
	Residual	269	168.88	0.63		
π	Quadrat	2	16.21	8.10	10.42	**.001**
	h_class	1	5.93	5.93	7.61	**.006**
	E/I	1	10.66	10.66	13.71	**.002**
	individual(quadrat)	31	91.99	2.98	3.81	**.001**
	quadrat*h_class	2	3.93	1.97	2.53	.084
	quadrat*E/I	2	9.94	4.97	6.39	**.005**
	h_class*E/I	1	4.27	4.27	5.49	**.018**
	individual(quadrat)*h_class	31	15.32	0.49	0.63	.938
	individual(quadrat)*E/I	31	26.90	0.87	1.11	.327
	quadrat*h_class*E/I	2	2.72	1.36	1.75	.163
	individual(quadrat)*h_class*E/I	31	17.44	0.56	0.72	.855
	Residual	269	209.26	0.78		

*df*, degrees of freedom; SS, sum of squares, MS, mean squares; Pseudo‐*F*: pseudo‐*F* statistics.

Bold text indicates *p*‐values <.05

**Figure 4:**
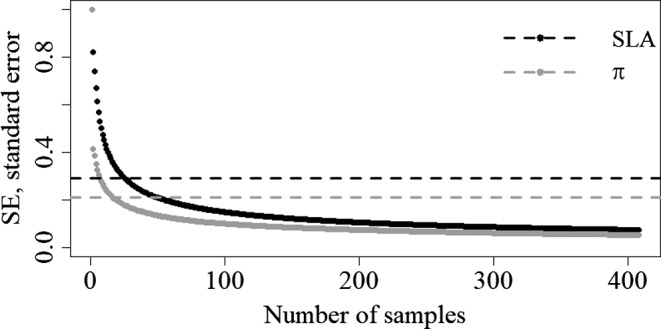
Relationship between number of samples considered and associated standard error (*SE*) of SLA (black points) and π (gray points)

**Figure 5:**
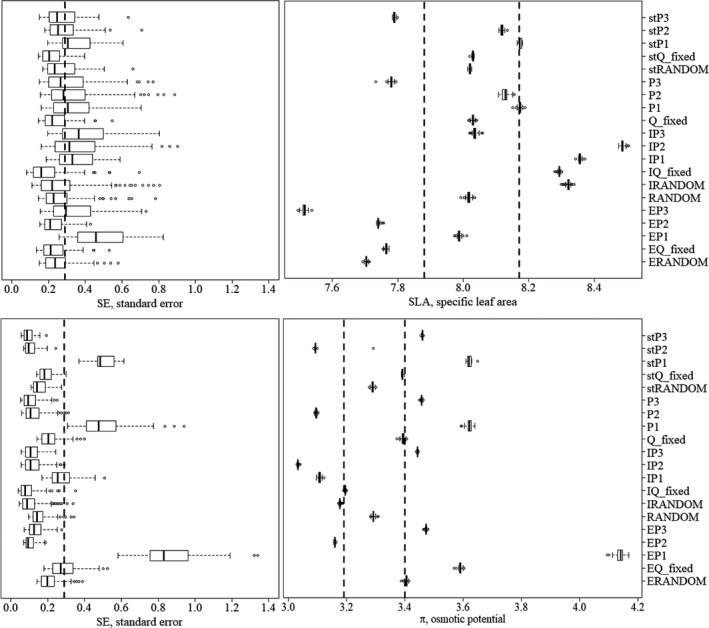
Median values, 25th and 75th percentiles of standard error (*SE*, left boxes) and median values, 25th and 75th percentiles of specific leaf area (SLA, mm^2^/mg, upper right box) and osmotic potential (π, ‐MPa, lower right box) calculated for each resampling strategy tested in this study. Dotted line in *SE* boxes indicates breakpoint values of *SE* of the two traits (0.29 for SLA and 0.22 for π), while dotted lines in upper and lower right boxes indicates 95% CI calculated for SLA and π

Figure 4 shows the relationship between the standard error, as a precision measurement, calculated on the two traits and the number of leaves considered. Figure 5 summarizes the mean standard error and mean values of the two traits obtained resampling data adopting the tested sampling strategy. Strategies were not considered representative of the sampled population if the mean values of SLA and π were out of CI range (Table 2) and/or *SE* were higher than *SE*_min_. Only four of the 15 tested resampling strategies satisfied these criteria: RANDOM, Q_fixed, stRANDOM, and stQ_fixed.

**Table 2:** Mean values and upper and lower CI at 95% of specific leaf area (SLA, mm^2^/mg) and osmotic potential (π, ‐MPa) calculated on the entire population

	SLA, mm^2^/mg	π, ‐MPa
Mean value	8.02	3.29
Upper CI (95%)	8.17	3.40
Lower CI (95%)	7.88	3.19

**Figure 6:**
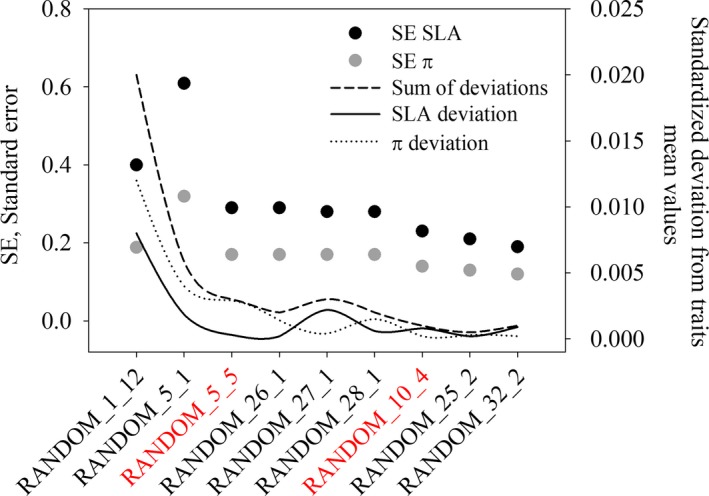
Standard error (*SE*) and deviations from mean values of specific leaf area (SLA, mm^2^/mg) and for osmotic potential (π, ‐MPa) in different sampling size of RANDOM sampling strategy. Sampling sizes in red represent the minimum (RANDOM_5_5) and optimal (RANDOM_10_4) sampling sizes to estimate SLA and π with desired precision and accuracy

RANDOM had the lowest deviation from the mean values of the two traits and were consequently considered the most accurate strategies to take into account trait's variability with respect to the whole set of sampled leaves. Figure 6 summarizes the statistics related to different sampling sizes within RANDOM family sampling strategies. The minimum size corresponded to 5 leaves taken randomly from 5 individuals (25 leaves in total, equal to the 6% of the whole set of sampled leaves), while the most accurate and feasible sampling size was 4 leaves taken randomly from 10 individuals (40 leaves in total, equal to the 10% of the whole set of sampled leaves; Figure 6).

## 4 DISCUSSION

The major goal of our analysis was to assess the sampling strategy that can adequately capture mean values of functional traits for a species/population, while maintaining information about traits’ variability and minimizing the sampling size and effort. In this perspective, we provided an analysis of the contribution of different factors, both spatial and biological, to the observed variability of two foliar functional traits, SLA and π. The factors tested in this study accounted for different proportion of the variance of the two traits, suggesting a different response of the traits to the different micro‐environmental conditions occurring through the canopy and within the study area. Several studies have reported that sources of traits’ variation depend on the vertical (height), horizontal (outer or inner branches), and azimuthal (aspect) position of the leaves and branches within the crown (Niinemets, Cescatti, & Christian, 2004). Considering the canopy's structure, light availability decreases moving from the top to the base and from external to internal portion of the crown (Niinemets, Keenan, & Hallik, 2015). SLA responds to this gradient, as it generally increases as light availability decreases (Poorter, Niinemets, Poorter, Wright, & Villar, 2009). A higher SLA could arise because of an increase in leaf area and/or a reduction in leaf biomass. Larger leaves improve the ability of the plant to intercept sun light and, consequently, the rate of photosynthesis. Alternatively, the reduction in biomass could allow plants to reduce the investment costs of shaded leaves (Nardini, Pedá, & Salleo, 2012). These mechanisms could explain why h_class and E/I resulted the main factors contributing to the variance of SLA (43.4% of the total variance of SLA).

Factors related to the canopy structure accounted for ~23% of the total variance of π. As mentioned above, light availability changes accordingly to canopy structure. Consequently, higher and external leaves intercept a higher amount of light, inducing stomatal aperture. Moreover, the wind speed is higher in the outer canopy, reducing the boundary layer resistance to water vapor diffusion from leaves to the atmosphere. All these factors lead to decreased water potential of leaves in the outer canopy, and the decrease in leaves π might help leaves to maintain turgor despite larger water losses. This could explain why factors h_class and E/I and their interaction significantly affected π variance. Interestingly, factors related to the spatial structure of the study area (quadrat, individual, and their interaction with other factors) accounted for the highest amount of variance of π (43.2%). This suggests that ITV of π is mostly spread among individuals rather than within individuals, contrary to SLA variance. The reason for this pattern may be related to the fact that the water status of a plant strongly depends on soil water availability (Binks et al., 2016). Different quadrats could have different soil water availability, because of heterogeneous soil structure or different competition for water with co‐occurring species. In the last years, different studies investigated traits variability and, in particular, Messier et al. (2010) assessed variability at different ecological scales of leaf dry matter content (LDMC) and leaf mass per area (LMA), the inverse of SLA. Patterns in ITV of LDMC and LMA were similar to those found by us in SLA but not for π. In fact, plot (which corresponds to quadrat in our study) accounted for the lowest amount of variance of SLA and trees (individuals in our study) accounted for ~20% of the total variability. Otherwise, as described above, the pattern of ITV of π was the opposite, as the main drivers of its variability were factors related to the study area spatial structure (e.g., quadrat and individuals). This difference between the soft traits (SLA, LMA, and LDMC) and the mechanistic trait (π) opens interesting questions about the patterns of ITV, encouraging future analyses including more mechanistic traits and more species.

The unequal contribution of the tested factors to the total variability of the two functional traits may imply that different traits should be sampled following different strategies. Over the last years, one of the most debated issues in trait‐based ecology has in fact regarded the choice of appropriate sampling strategy (Baraloto et al., 2010; Carmona, Rota, Azcárate, & Peco, 2015; Paine, Baraloto, & Díaz, 2015). Trees that belong to the same species and inhabit the same population of natural origin can show strong genetic and phenotypic differences (Messier et al., 2010). The phenotype is in turn heavily influenced by the macro‐ and micro‐environmental conditions and by the relationships with the neighboring trees (competition and/or co‐operation) (Abakumova, Zobel, Lepik, & Semchenko, 2016). To the best of our knowledge, no study has investigated the ITV_WI_ and only few reports have provided information on sampling strategies based on measurements of precision (Cornelissen et al., 2003; Pérez‐Harguindeguy et al., 2013). RANDOM, stRANDOM, Q_fixed, and stQ_fixed resulted the strategies that better estimated the mean values of the traits with an adequate precision (*SE* lower than *SE* profiles flex points, Figure 2) and dispersion of probability distribution (CV higher than CV profiles flex points, Fig. S2). Of these four strategies, the most accurate was RANDOM strategy, as it minimized deviations from traits mean value. The minimum size required to accurately and precisely estimate the two traits was represented by RANDOM_5_5 (sampling 5 random individuals from the 3 quadrats and 5 leaves per individual). Increasing sampling size resulted in a decrease of *SE* (increase in precision), but this trend was not consistent for S (Figure 6). This means that an increase in the sampling effort not always translates in better accuracy. The most significant drop of trait S and sum of S was at RANDOM_10_4 (4 leaves from 10 random individuals), which could be considered as a good compromise between sampling effort and precision of the measurements of the two traits rather than RANDOM_32_2, RANDOM _26_1, RANDOM _27_1, and RANDOM _28_1. Our findings partially confirm results from Baraloto et al. (2010) and Carmona et al. (2015), as they reported that strategy equivalent to the RANDOM strategy was the most precise and accurate to estimate species mean trait values and community weighted mean (CWM). They also concluded that sampling at least one individual per species could be a good compromise to describe species mean traits value. On the contrary, in our study, sampling all possible leaves of one individual (RANDOM_1_12) produced traits estimates far under the desired level of precision and accuracy (Figure 6). This difference could be due to the different spatial and ecological scales of our analysis versus Baraloto et al. (2010) and Carmona et al. (2015) analyses. Our analysis was conducted at local scale, where an accurate estimate of the variance of trait values is more important than at broader scale (Baraloto et al., 2010). Hence, we suggest including the minimum and preferred sampling size provided here in studies aimed at highlighting differences in species/communities at local scale, while the approach proposed by Baraloto et al. (2010) and Carmona et al. (2015) could be adopted in analyses at broader scales. We also compared our results with those included in standard protocol proposed by Pérez‐Harguindeguy et al. (2013). They provide a minimum and a preferred number of individuals and leaves per individual based on the calculation of CV of each trait considered in different studies and on common practice. For SLA, they recommend sampling 5 leaves from 5 individuals (minimum) or 4 leaves from 10 individuals (preferred). However, they also suggested sampling “sun leaves” (leaves positioned in the outer canopy stratum) for the measurements of SLA. We interpreted this suggestion in two possible ways. Outer canopy could be represented by leaves in the external stratum of the canopy at any height (see cor, cor_min, per, per_min strategies, Table S2) or by leaves in the upper and external stratum of the canopy (see cor_b, cor_min_b, per_b, per_min_b strategies, Table S2). The mean values of the two traits calculated for each resampling strategy listed above (Table S2) lie outside the 95% CI range (Table 2) calculated on the whole dataset. Hence, SLA values measured following standardized protocols results significantly different from SLA mean values measured on the entire dataset. Sampling “sun leaves” was proposed in the past to control variation of leaf traits to avoid shading bias (Messier et al., 2010). However, our data suggest that the exclusion of other canopy strata could produce underestimation not only of mean traits values, but also of traits variability, as CV measured only on external leaves was lower than minimum CV (Table S3, Fig. S3). Such underestimation can lead to underestimations of physiological leaf processes that change as a function of leaf surface such as transpiration rate, gas exchanges, and photosynthesis rate (Poorter et al., 2009). Moreover, Keenan & Niinemets (2016) demonstrated that most of the measurements of SLA in current available databases are strongly biased by shading effect. In fact, leaves experience strong changes in terms of light availability during their development (i.e., light gradients within a canopy or across gap‐understory continua). Consequently, the dichotomy between “sun” and “shade” leaves could be considered ambiguous. As previously proposed by Messier et al. (2010), it would be preferable to distinguish between measurements of SLA only considering sun leaves (SLA_max_ or SLA_sun_) from measurements considering the whole canopy (SLA_tree_). In the light of the above, we recommend sampling leaves considering all the strata composing the canopy to estimate correctly the studied traits values, especially when interested in assessing traits variability at species level.

## 5 CONCLUSION

Our analysis confirms the role of ITV in determining leaf functional traits variability. Moreover, we demonstrate that the spatial structure of the canopy could significantly affect traits variability. Interestingly, different factors accounted for different proportion of the variability of the two traits, suggesting that different strategies for different traits could be implemented during sampling surveys.

We also provided practical advices to optimize sampling procedures of functional traits: a minimum (five leaves from five individuals) and optimal (four leaves from 10 individuals) sample size based on measurements of precision, partially confirming sampling sizes previously proposed by literature. The results presented here should encourage future analysis involving different traits, in order to get global insights into ITV as based on multiple trait analysis.

## CONFLICT OF INTEREST

We declare the absence of any conflicts of interests at the time of submission.

## AUTHOR CONTRIBUTIONS

FP, AN, and GB conceived and designed the experiment; FP, CP, TS, RA, and GB collected the data; FP, CP, and GB analyzed the data; FP, AN, and GB wrote the manuscript, with the contribution of all authors.

## DATA ACCESSIBILITY

The complete dataset has been deposited in Dryad: https://doi.org/10.5061/dryad.6362p
