# Supplementary material for: Sampling intraspecific variability in leaf functional traits: Practical suggestions to maximize collected information
Source: Ecol Evol. 2017 Nov 21;7(24):11236–45. doi: 10.1002/ece3.3617 (PMC5743657; doi:10.1002/ece3.3617)
Supplement: Supplementary file 1 [file ECE3-7-11236-s001.doc]

**Supporting Information**

Table captions

Table S1. List of virtual resampling strategies tested.

Table S2. Resampling strategies following standardized protocol suggested criteria (Cornelissen et al., 2003; Pérez-Harguindeguy et al., 2013).

Table S3. Mean, SE and CV values of specific leaf area (SLA, mm^2^ mg^-1^) and osmotic potential (π, -MPa) calculated for each resampling strategies listed in Table 1 and Table 2. Bold text indicates the most precise resampling strategies within the entire pool of strategies tested.

Figure legends

Fig. S1. Map of the three study areas with the highest density of *Q. ilex*, where samples were collected.

Fig. S2. Relationship between number of samples considered and associated coefficient of variation (CV) of SLA (black points) and π (grey points).

Fig. S3. Median values, 25^th^ and 75^th^ percentiles of coefficient of variation (CV) of specific leaf area (SLA, mm^2^ mg^-1^) and osmotic potential (π, -MPa) calculated for each resampling strategy tested in this study. Dotted lines indicates maximum CV of the two traits calculated on the whole dataset.

| *Resampling strategies* | *Description* |
| --- | --- |
| **RANDOM** | . “i” individuals from all the 34 sampled are chosen randomly, then “f” leaves per individual are randomly sampled) |
| **Q_fixed** | a fixed number of individuals per quadrat (“f” leaves per individual are randomly sampled) |
| **P1** | only individuals from quadrat n°1 were randomly chosen |
| **P2** | only individuals from quadrat n°2 were randomly chosen |
| **P3** | only individuals from quadrat n°3 were randomly chosen |
| **stRANDOM** | Hierarchical stratified = the choice of individuals is the same of previous resampling strategies, but leaves were sistematically chosen from the canopy stratum defined by height class (“a” or “b”) and external (E) or internal (I) foliage. |
| **stQ_fixed** |
| **stP1** |
| **stP2** |
| **stP3** |
| **ERANDOM** | The choice of individuals is the same of first 5 resampling strategies, but leaves were randomly selected only from external (E) foliage. |
| **EQ_fixed** |
| **EP1** |
| **EP2** |
| **EP3** |
| **IRANDOM** | The choice of individuals is the same of first 5 resampling strategies, but leaves were randomly selected only from internal (I) foliage. |
| **IQ_fixed** |
| **IP1** |
| **IP2** |
| **IP3** |

Table S1

| ***Resampling strategies*** | | ***Description*** |
| --- | --- | --- |
| **Cornelissen et al. (2003)** | cor | Preferred number of leaves taken randomly from external canopy (E). |
|  | cor_b | Preferred number of leaves taken randomly from external (E) and upper canopy (b). |
|  | cor_min | Minimal number of leaves taken randomly from external canopy (E). |
|  | cor_min_b | Minimal number of leaves taken randomly from external (E) and upper canopy (b). |
| **Pérez-Harguindeguy et al. (2013)** | per | The same as the previous sampling strategies but referred to preferred and minimal number of leaves to be sampled by Pérez-Harguindeguy et al. (2013) |
|  | per_b |  |
|  | per_min |  |
|  | per_min_b |  |

Table S2

| *Resampling strategy* | **SLA, mm^2^ mg^-1^** | | | **π, -MPa** | | |
| --- | --- | --- | --- | --- | --- | --- |
|  | *Mean* | *SE* | *CV* | *Mean* | *SE* | *CV* |
| **RANDOM** | **8.02** | **0.27** | **0.18** | **3.29** | **0.16** | **0.25** |
| **Q_fixed** | **8.03** | **0.25** | **0.17** | **3.39** | **0.21** | **0.35** |
| P1 | 8.17 | 0.33 | 0.14 | 3.62 | 0.48 | 0.47 |
| P2 | 8.12 | 0.30 | 0.19 | 3.10 | 0.12 | 0.19 |
| P3 | 7.78 | 0.28 | 0.17 | 3.46 | 0.09 | 0.13 |
| **stRANDOM** | **8.02** | **0.28** | **0.18** | **3.29** | **0.16** | **0.25** |
| **stQ fixed** | **8.03** | **0.22** | **0.17** | **3.39** | **0.19** | **0.35** |
| stP1 | 8.17 | 0.34 | 0.14 | 3.62 | 0.50 | 0.46 |
| stP2 | 8.12 | 0.30 | 0.19 | 3.10 | 0.11 | 0.19 |
| stP3 | 7.79 | 0.28 | 0.17 | 3.46 | 0.10 | 0.13 |
| ERANDOM | 7.70 | 0.29 | 0.17 | 3.41 | 0.22 | 0.31 |
| EQ_fixed | 7.76 | 0.24 | 0.17 | 3.60 | 0.29 | 0.45 |
| EP1 | 7.99 | 0.49 | 0.15 | 4.14 | 0.88 | 0.52 |
| EP2 | 7.74 | 0.33 | 0.17 | 3.16 | 0.14 | 0.19 |
| EP3 | 7.51 | 0.34 | 0.15 | 3.47 | 0.14 | 0.14 |
| IRANDOM | 8.32 | 0.26 | 0.18 | 3.18 | 0.10 | 0.18 |
| IQ_fixed | 8.29 | 0.19 | 0.17 | 3.19 | 0.09 | 0.21 |
| IP1 | 8.36 | 0.36 | 0.11 | 3.11 | 0.28 | 0.23 |
| IP2 | 8.49 | 0.36 | 0.18 | 3.03 | 0.12 | 0.17 |
| IP3 | 8.04 | 0.40 | 0.17 | 3.44 | 0.12 | 0.11 |
| cor | 7.69 | 0.30 | 0.17 | 3.40 | 0.24 | 0.30 |
| cor_b | 6.98 | 0.21 | 0.13 | 3.23 | 0.15 | 0.21 |
| cor_min | 7.71 | 0.35 | 0.17 | 3.40 | 0.25 | 0.25 |
| cor_min_b | 6.98 | 0.31 | 0.12 | 3.23 | 0.21 | 0.18 |
| per | 7.70 | 0.22 | 0.18 | 3.40 | 0.19 | 0.33 |
| per_b | 6.99 | 0.17 | 0.13 | 3.23 | 0.13 | 0.21 |
| per_min | 7.70 | 0.27 | 0.17 | 3.41 | 0.22 | 0.30 |
| per_min_b | 7.00 | 0.24 | 0.13 | 3.23 | 0.17 | 0.20 |

Table S3


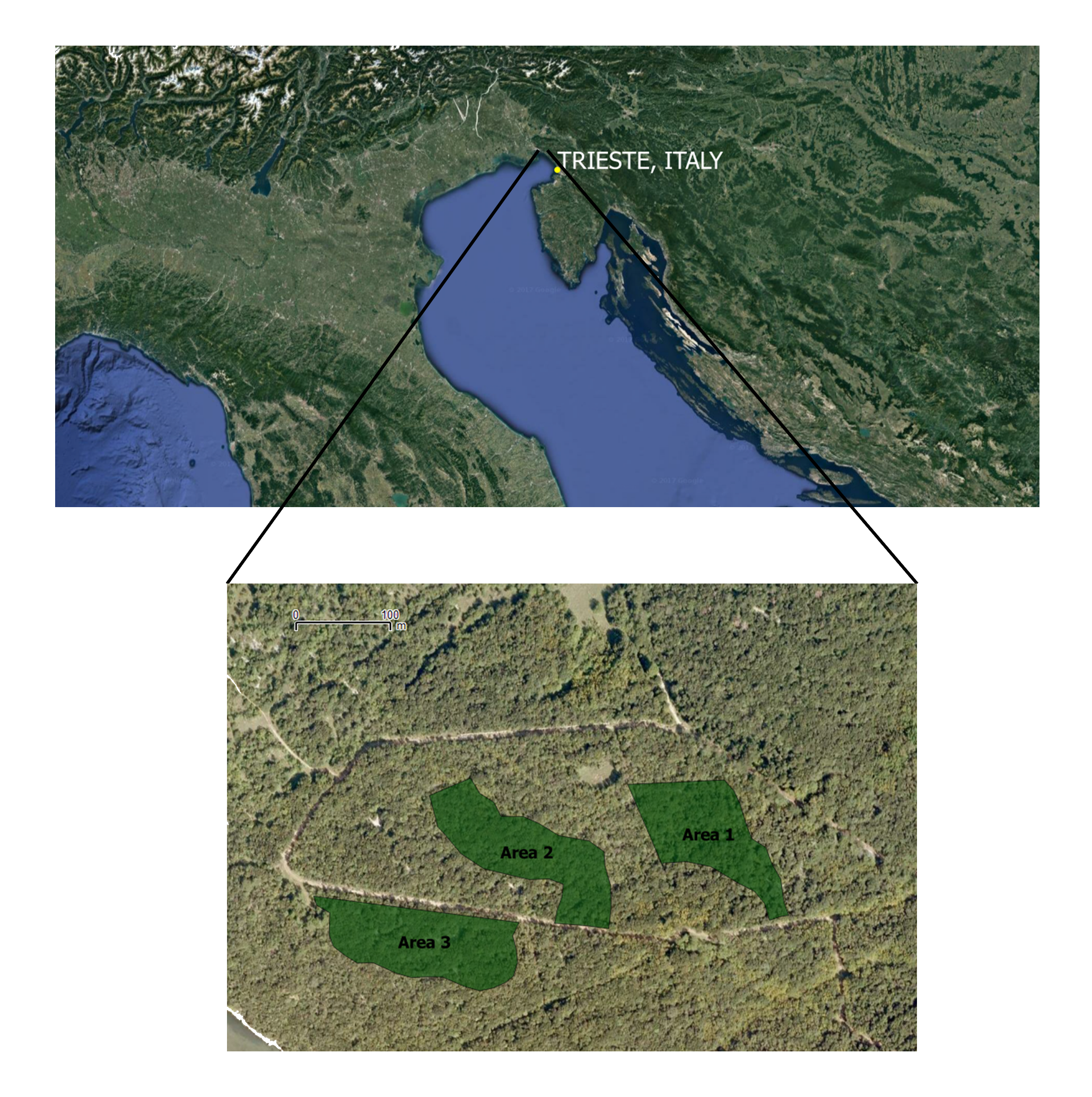


Fig. S1


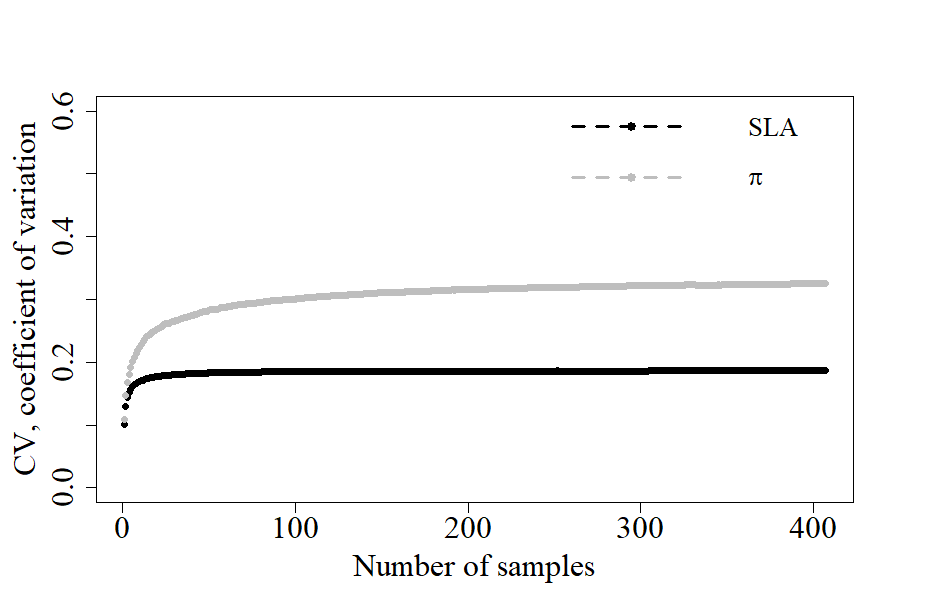


Fig. S2


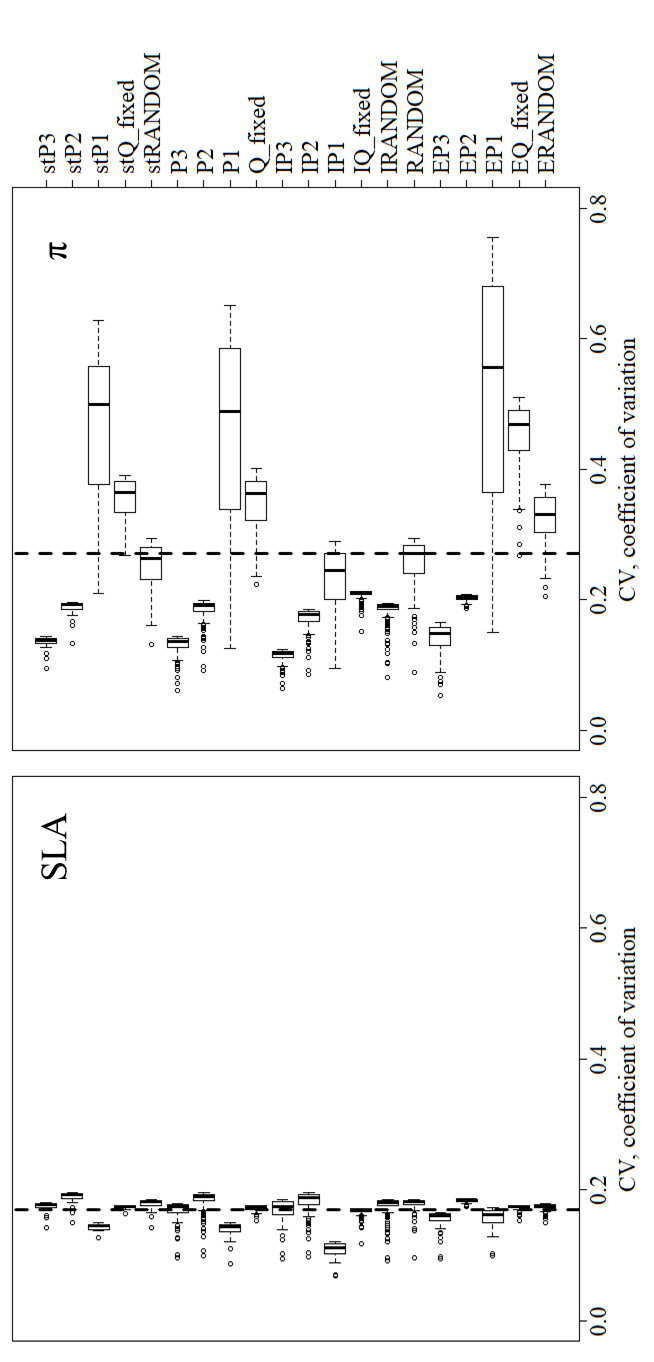


Fig. S3
